# The structure of Tim50(164–361) suggests the mechanism by which Tim50 receives mitochondrial presequences

**DOI:** 10.1107/S2053230X15013102

**Published:** 2015-08-25

**Authors:** Jingzhi Li, Bingdong Sha

**Affiliations:** a Department of Cell, Developmental and Integrative Biology (CDIB), University of Alabama at Birmingham, Birmingham, AL 35294, USA

**Keywords:** Tim50, preprotein transport, IMS domain

## Abstract

Mitochondrial preproteins are transported through the translocase of the outer membrane (TOM) complex. Tim50 and Tim23 then transfer preproteins with N-terminal targeting presequences through the intermembrane space (IMS) across the inner membrane. The crystal structure of the IMS domain of Tim50 [Tim50(164–361)] has previously been determined to 1.83 Å resolution. Here, the crystal structure of Tim50(164–361) at 2.67 Å resolution that was crystallized using a different condition is reported. Compared with the previously determined Tim50(164–361) structure, significant conformational changes occur within the protruding β-hairpin of Tim50 and the nearby helix A2. These findings indicate that the IMS domain of Tim50 exhibits significant structural plasticity within the putative presequence-binding groove, which may play important roles in the function of Tim50 as a receptor protein in the TIM complex that interacts with the presequence and multiple other proteins. More interestingly, the crystal packing indicates that helix A1 from the neighboring monomer docks into the putative presequence-binding groove of Tim50(164–361), which may mimic the scenario of Tim50 and the presequence complex. Tim50 may recognize and bind the presequence helix by utilizing the inner side of the protruding β-hairpin through hydrophobic interactions. Therefore, the protruding β-hairpin of Tim50 may play critical roles in receiving the presequence and recruiting Tim23 for subsequent protein translocations.

## Abstract 2

The Tim50 crystal structure indicates that the IMS domain of Tim50 exhibits significant structural plasticity within the putative presequence-binding groove.

## 1. Introduction   

The mitochondrion contains a large number of proteins. However, the mitochondrion can only synthesize a few proteins by itself (Sickmann *et al.*, 2003 ▸; Gray *et al.*, 1999 ▸). The majority of mitochondrial proteins are translated in the cytosol and transported to the mitochondrion by a multiprotein complex: the translocase of the outer membrane complex (TOM) and the translocase of the inner membrane complex (TIM) (Neupert & Herrmann, 2007 ▸). More than half of the mitochondrial preproteins are synthesized with N-terminal presequences that form positively charged amphipathic helices that target the preproteins to the mitochondria. The preproteins are imported by the general translocase of the outer membrane (TOM) complex and the presequence translocase of the inner membrane (TIM23) complex (Abe *et al.*, 2000 ▸; Chacinska *et al.*, 2009 ▸; Wu & Sha, 2006 ▸; Alder *et al.*, 2008 ▸; Vögtle *et al.*, 2009 ▸). After passing through the TOM complex, preproteins with N-terminal targeting presequences are received by Tim50, an essential member of the TIM23 complex, in the intermembrane space (IMS; Geissler *et al.*, 2002 ▸; Yamamoto *et al.*, 2002 ▸; Mokranjac *et al.*, 2003 ▸; Rahman *et al.*, 2014 ▸). Within the TIM23 complex, the C-terminal half of the Tim23 protein forms the transmembrane channel across the mitochondrial inner membrane. The IMS domain of Tim50 interacts with the N-terminal IMS domain of Tim23 to deliver the preproteins to Tim23 (Schulz *et al.*, 2011 ▸). Tim50 also functions as a hub protein to interact with other TIM23 complex members such as Tim21 to regulate the opening and closure of the Tim23 transmembrane channel (Chacinska *et al.*, 2005 ▸; Meinecke *et al.*, 2006 ▸; Lytovchenko *et al.*, 2013 ▸).

The IMS domain of Tim50 contains a trypsin-resistant core domain which contains residues 164–361. The crystal structure of Tim50(164–361) has been determined to a resolution of 1.83 Å (Qian *et al.*, 2011 ▸). The crystal structure of Tim50(164–361) contains a large groove as a putative binding site for the presequences and a protruding β-hairpin. It has been demonstrated that the protruding β-hairpin is crucial for the interaction of Tim50 with Tim23, suggesting a cooperative function of these two essential TIM23 proteins in preprotein import. It has been reported that the N-terminal targeting presequence constitutes an amphipathic helix (Abe *et al.*, 2000 ▸). The putative presequence-binding groove identified in the Tim50(164–361) structure is large enough to accommodate an α-helix formed by the presequence. However, it is not clear how the preprotein receptor Tim50 interacts with the pre­sequence helix at the molecular level.

As the preprotein receptor in the intermembrane space, Tim50 has the ability to interact with a large number of different presequences. In order to accommodate different presequences, it is possible that Tim50 may exhibit conformational flexibility at the putative presequence-binding groove. This study presents structural evidence to show that the putative presequence-binding groove of Tim50 exhibits significant plasticity to interact with different presequences. Interestingly, the crystal packing in the newly solved Tim50(164–361) structure provides information to reveal the mechanism by which the IMS domain of Tim50 specifically recognizes and interacts with the presequence helix.

## 2. Methods   

*Saccharomyces cerevisiae* Tim50(164–361) was expressed and purified as described by Qian *et al.* (2011 ▸). The crystals were grown using the hanging-drop vapor-diffusion method in the condition 100 m*M* MES pH 6.0, 1.6 *M* MgSO_4_ (Table 1 ▸). The crystals were needle-shaped, with dimensions of 0.1 × 0.1 × 0.5 mm. Data were collected from crystals cooled in mother liquor containing 20% glycerol. The crystals diffracted X-rays to 2.67 Å resolution on the SER-CAT beamline at APS (Table 2 ▸). These newly obtained crystals did not diffract as well as those produced previously (Qian *et al.*, 2011 ▸), possibly owing to the fact that a larger number of Tim50(164–361) molecules were present in the asymmetric unit in the newly obtained crystals. 100 images of diffraction data with an oscillation of 1° were utilized in data processing. The diffraction data were reduced and integrated using *HKL*-3000 (Minor *et al.*, 2006 ▸). The atomic coordinates of the Tim50(164–361) structure (PDB entry 3qle; Qian *et al.*, 2011 ▸) were used as a search model in the molecular-replacement method using *Phaser* (McCoy *et al.*, 2007 ▸). The model was manually built by *Coot* (Emsley & Cowtan, 2004 ▸). Refinement was carried out using *REFMAC* (Vagin & Teplyakov, 2010 ▸) from the *CCP*4 suite (Winn *et al.*, 2011 ▸). The coordinates and structure factors have been deposited in the PDB with accession code 4qqf.

## 3. Results and discussion   

In this study, we describe a crystal structure of yeast Tim50(164–361) which is significantly different from that which we reported previously (Qian *et al.*, 2011 ▸). The structural data support the proposal that the Tim50 putative presequence-binding groove may exhibit significant structural flexibility to recognize and interact with various preproteins for mitochondrial biogenesis. The data also illustrate the mechanism by which Tim50 can specifically recognize and interact with the amphipathic presequence helix.

**Figure 1:**
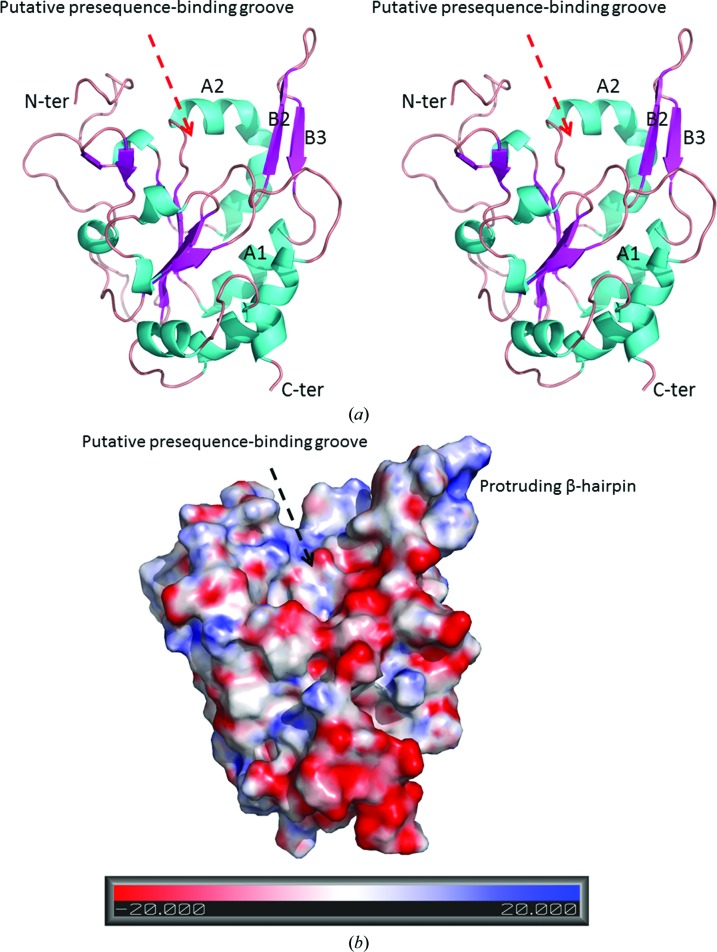
The crystal structure of Tim50_new. (*a*) The Tim50_new structure is shown in ribbon representation as a stereoview. The N-terminus and C-terminus of the molecule are labeled. The secondary structures A1, A2, B2 and B3 are labeled. The putative presequence-binding groove is indicated by an arrow. The Tim50 molecule shown in this figure is monomer *E* in the asymmetric unit of Tim50_new, which belongs to group I. (*b*). The Tim50_new structure is shown in surface potential representation. The orientation of the Tim50_new molecule is similar to that in (*a*). The scale for the surface potential is shown below the figure. The figures in this paper were all generated using *PyMOL*.

The crystal structure of *S. cerevisiae* Tim50(164–361) determined in this study is named Tim50_new and the crystal structure of Tim50(164–361) described previously is termed Tim50_old (Qian *et al.*, 2011 ▸). The structure of Tim50_new was determined to 2.67 Å resolution by the molecular-replacement method using Tim50_old as the search model (Tables 1 ▸ and 2 ▸). In the Tim50_new structure, electron density is visible for residues 177–356. In Tim50_new, the Tim50 IMS-domain molecule forms a monomer and consists of five α-helices (A1–A5) and nine β-strands (B1–B9) (Fig. 1 ▸). The core of the structure is constituted by a parallel β-sheet formed by B1, B4, B5, B8 and B9. A β-hairpin that protrudes out of the Tim50 molecular surface by ∼15 Å is formed by B2 and B3 and the short loop between B2 and B3 (Fig. 1 ▸). Close to the protruding β-hairpin, Tim50_new contains a large groove which has been hypothesized to be the presequence-binding site (Fig. 1 ▸). Six monomers are present in one asymmetric unit of Tim50_new, while only one monomer is present in the asymmetric unit of Tim50_old.

**Figure 2:**
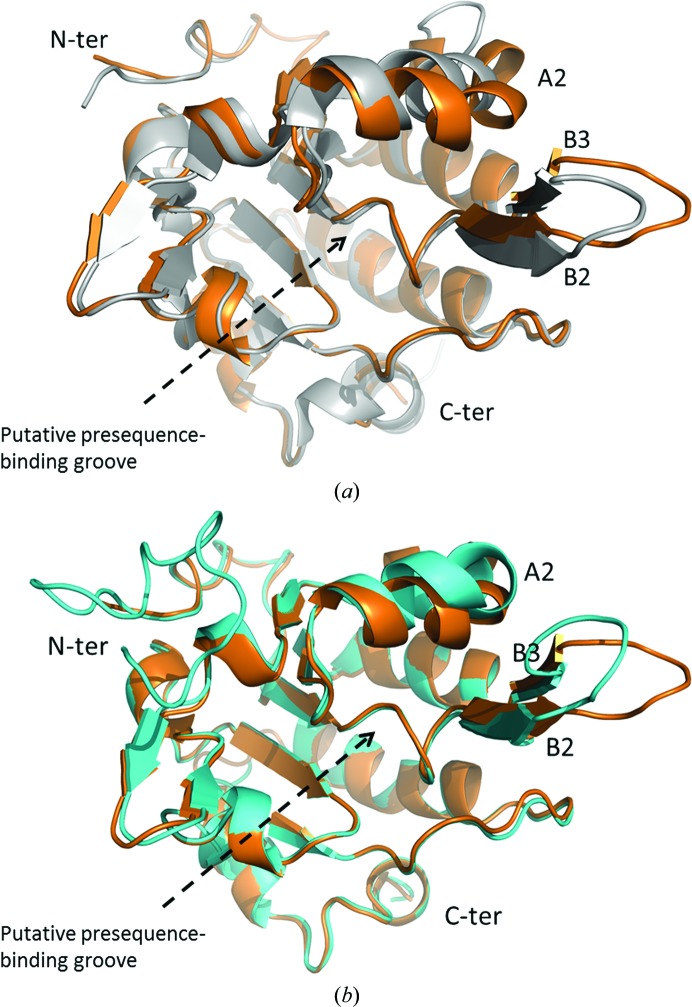
The protruding β-hairpin and the nearby helix A2 exhibit structural plasticity in the Tim50 molecule. (*a*) The Tim50_new structure in gold is superimposed with the Tim50_old structure in silver. The Tim50 molecules in this figure are rotated about 90° around the horizontal axis from the orientation in Fig. 1 ▸. The putative presequence-binding groove faces the reader in this figure. The secondary structures A2, B2 and B3 are labeled. The N-terminus and C-terminus of Tim50 are labeled. The Tim50_new structure shown here is monomer *E* in the asymmetric unit, which belongs to group I. (*b*) Structure superimposition of Tim50_new monomers from groups I and II. The group I monomer *E* is in gold and the group II monomer *A* is in cyan. The secondary structures A2, B2 and B3 are labeled. The N-terminus and C-terminus of Tim50 are labeled. The Tim50 molecules in this figure are in similar orientations to those in (*a*). The putative presequence-binding groove is labeled.

When the Tim50_new structure is compared with that of Tim50_old, the majority of the molecules superimpose quite well. However, significant conformational changes occur in the protruding β-hairpin formed by B2 and B3 and in the nearby helix A2 (Fig. 2 ▸ *a*). The main body of Tim50_new excluding the protruding β-hairpin and helix A2 can be superimposed well with its counterpart in Tim50_old, with a root-mean-square deviation (r.m.s.d) of 0.550 Å for the main-chain atoms. In contrast, the tip of the protruding β-hairpin shifts away from the main body of the molecule by 4.5 Å in the Tim50_new structure when compared with that in the Tim50_old structure (Fig. 2 ▸ *a*). Meanwhile, the nearby helix A2 moves by ∼5 Å towards the protruding β-hairpin (Fig. 2 ▸ *a*). Moreover, helix A2 in Tim50_new contains two more turns than that in Tim50_old. Because the protruding β-hairpin and helix A2 form one side of the putative presequence-binding groove of Tim50, the conformational changes in Tim50_new may significantly alter the physical properties of the groove. These structural observations indicate that the IMS domain of Tim50 exhibits significant structural plasticity within the putative presequence-binding groove, which may play important roles in the function of Tim50 as a receptor protein in the TIM23 complex that interacts with various pre­sequences. It is reasonable that the Tim50 putative presequence-binding groove has the capability to adjust its own conformation to accommodate presequences with various sizes, hydrophobicities and other physical properties.

The structural plasticity of the Tim50 putative presequence-binding groove can be further illustrated by comparing the six monomer structures within one asymmetric unit of Tim50_new. The six monomers within one asymmetric unit of Tim50_new are termed molecules *A*–*F*. When the structures of the six molecules were carefully examined, we found that the six molecules could be divided into two groups. Molecules *B*, *C* and *E* share very similar structures throughout the structure and belong to group I. On the other hand, molecules *A*, *D* and *F* adopt a different conformation within the protruding β-hairpin and helix A2 from those in group I and belong to group II. When the structures of group I members are superimposed with those of group II, it is clear that the main body of the structures is very similar between the members of the two groups. However, the protruding β-hairpin and helix A2 show significant differences between the members of the two groups (Fig. 2 ▸ *b*). The root-mean-square deviation (r.m.s.d) for the main-chain atoms for the protruding β-hairpin and helix A2 is ∼2 Å when group I and II monomers are superimposed, indicating significant conformational changes within the putative presequence-binding groove. The protruding β-hairpin and helix A2 in members of groups I and II from Tim50_new and in Tim50_old show three distinct conformations. Therefore, our structural evidence from comparing the Tim50_new and Tim50_old structures and from comparing the monomers within the asymmetric unit of Tim50_new indicate that the IMS domain of Tim50 exhibits significant structural plasticity around the putative presequence-binding groove. The protruding β-hairpin and helix A2 within the IMS domain of Tim50 can adopt multiple conformations, while the main body of Tim50(164–361) remains in one stable conformation.

**Figure 3:**
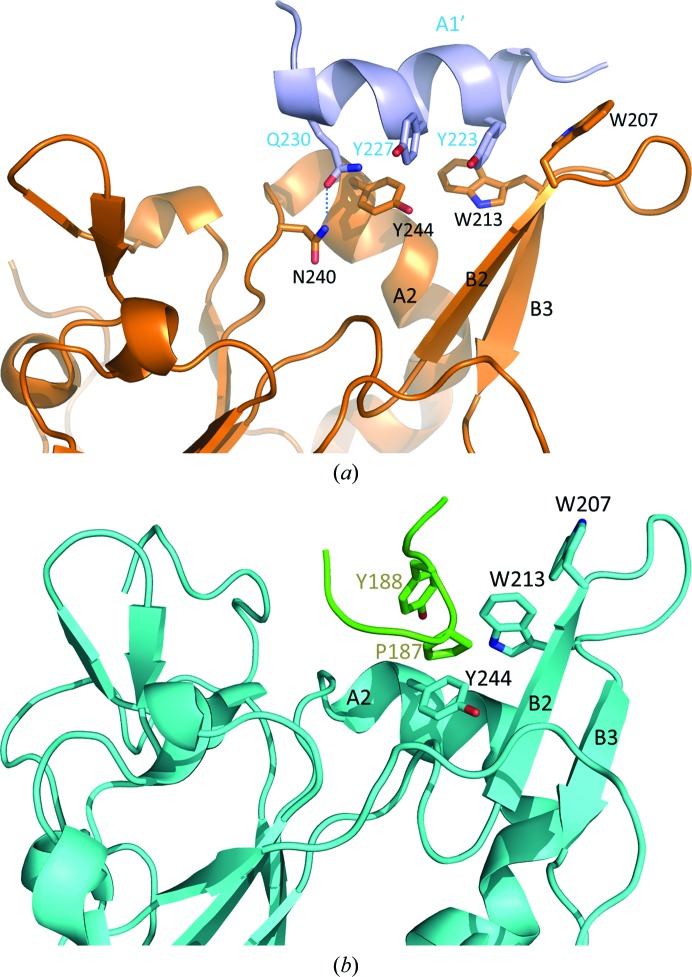
The putative presequence-binding grooves of Tim50_new molecules are occupied by secondary structures from neighboring molecules generated by crystal packing. (*a*) Helix A1 from the neighboring molecule (monomer *A*) is docked into the putative presequence-binding groove of monomer *E* of the Tim50_new structure primarily by hydrophobic interactions. Monomer *E* of Tim50_new belongs to group I and is shown in gold. Helix A1 from the neighboring molecule is shown in light blue and is labeled A1′. The secondary structures A2, B2 and B3 of monomer *E* are labeled. The residues Tyr223, Tyr227 and Gln230 from A1′, residues Trp207 and Trp213 from B2 and B3 and residues Asn240 and Tyr244 from A2 that are involved in the interactions are labeled. The hydrogen bond between Gln230 from A1 and Asn240 from A2 is indicated by a dotted line. (*b*) The N-terminal proline-rich loop from the neighboring molecule is inserted into the putative presequence-binding groove of monomer *A* of the Tim50_new structure by hydrophobic interactions. Monomer *A* of the Tim50_new structure belongs to group II and is shown in cyan. The N-­terminal proline-rich loop from the neighboring molecule is shown in green. The residues Pro187 and Tyr188 from the N-terminal proline-rich loop, residues Trp207 and Trp213 from B2 and B3 and residue Tyr244 from A2 that are involved in the interactions are labeled.

It is interesting to note that the protruding β-hairpin and helix A2 exhibit different conformations even in structures from the same crystallization conditions. To further understand the mechanism by which the group I and group II members of Tim50_new adopt different conformations, we examined the neighboring molecules of the group I and group II members generated by crystal packing. We reasoned that the neighbouring environment of Tim50(164–361) may play an important role in the conformation that Tim50(164–361) adopts. Surprisingly, for members of both group I and group II the putative presequence-binding grooves are occupied by secondary structures from the neighboring monomers. For group I members helix A1 from the neighboring monomer is docked into the putative presequence-binding groove, and for group II members the N-terminal proline-rich loop from the neighboring monomer is inserted into the putative pre­sequence-binding groove (Fig. 3 ▸). In sharp contrast, when we examined the crystal packing in the Tim50_old structure, the putative presequence-binding groove is virtually empty (data not shown). Therefore, it is likely that Tim50_old represents the presequence-free conformation of the IMS domain of Tim50 and the Tim50_new corresponds to the presequence-binding conformation of the IMS domain of Tim50. The structural differences between the members of group I and group II within one asymmetric unit of the Tim50_new structure suggests that the putative presequence-binding groove of the IMS domain of Tim50 may adopt different conformations to accommodate various presequences.

For the group I members of Tim50_new, the putative presequence-binding groove is occupied by helix A1 from the neighboring molecule generated through crystal packing (Fig. 3 ▸ *a*). Helix A1 from the neighboring molecule lies between the protruding β-hairpin and helix A2 and extends along the long dimension of the putative presequence-binding groove of Tim50(164–361). Helix A1 from the neighboring molecule is docked into the putative presequence-binding groove primarily *via* hydrophobic interactions. Tyr223 from helix A1 of the neighbouring molecule makes extensive hydrophobic interactions with Trp207 and Trp213 from the protruding β-hairpin. Trp207 and Trp213 are located on the same side of the β-hairpin and swing in opposite directions to accommodate Tyr223 from the neighboring molecule. Moreover, Tyr227 from helix A1 of the neighboring molecule makes a hydrophobic interaction with Tyr244 from helix A2. Gln230 from helix A1 of the neighboring molecule forms a hydrogen bond to Asn240 from helix A2. The interaction between the putative presequence-binding groove from the group I monomer and helix A1 from the neighboring monomer represents the sole contact between these two monomers.

It has been reported that the N-terminal mitochondrial targeting presequence forms an amphipathic helix to interact with the Tom20 receptor in the TOM complex to initiate protein translocation (Abe *et al.*, 2000 ▸). The association between the presequence and the Tom20 receptor is mediated through hydrophobic interactions. We propose that helix A1 from the neighboring molecule may mimic the presequence to interact with the Tim50 receptor in the TIM23 complex within the intermembrane space. Similar to the nature of the binding between the presequence and Tom20, Tim50 also interacts with the hydrophobic side of the presequence helix.

For the group II members of Tim50_new, the presequence-binding pocket is occupied by the N-terminal proline-rich loop from the neighboring monomer (Fig. 3 ▸ *b*). The proline-rich loop forms a sharp turn and interacts with the putative presequence-binding groove mainly through hydrophobic interactions. Pro187 and Tyr188 from the proline-rich loop make hydrophobic interactions with Trp207 and Trp213 from the protruding β-hairpin and Tyr244 from helix A2 (Fig. 3 ▸ *b*).

Our data suggest that the protruding β-hairpin and the nearby helix A2 play important roles in recognizing and interacting with the presequences of mitochondrial pre­proteins. The large hydrophobic residues Trp207 and Trp213 are both located on the inner side (facing towards the putative presequence-binding groove) of the protruding β-hairpin and make this side of the β-hairpin quite hydrophobic. It has been reported that the protruding β-hairpin represents the largest conserved area on the Tim50 molecular surface (Qian *et al.*, 2011 ▸). Sequence alignment of Tim50 showed that Trp207 and Trp213 were very well conserved among various species (Qian *et al.*, 2011 ▸). It is likely that the inner side of the protruding β-hairpin of the IMS domain of Tim50 is responsible for recognizing the presequence helix through hydrophobic interactions. The structural plasticity of the protruding β-hairpin and the nearby helix A2 may facilitate the accommodation of presequences with various sizes by Tim50.

Structural and mutagenetic analyses have suggested that the conserved residues Arg214 and Lys217 of Tim50 might be involved in interaction with the IMS domain of Tim23 (Qian *et al.*, 2011 ▸). Interestingly, residues Arg214 and Lys217 are located on the outer side (facing away from the putative presequence-binding groove) of the protruding β-hairpin. Therefore, the protruding β-hairpin may play critical roles in the functions of Tim50. The inner side of this β-hairpin is responsible for recognizing the hydrophobic surfaces of the presequence helix, while the outer side of the β-hairpin is involved in recruiting Tim23 for subsequent protein trans­location. Moreover, the protruding β-hairpin of Tim50 processes significant structural flexibility that facilitates its binding to the presequence and the IMS domain of Tim23. Interestingly, NMR spectroscopic analysis suggested that the IMS domain of Tim23 was also intrinsically flexible (de la Cruz *et al.*, 2010 ▸). The IMS domain of Tim23 interacts with Tim50 at a site which is in close proximity to the presequence-binding site of Tim50, leading to the hypothesis that Tim50 and Tim23 form a composite presequence-binding pocket. This also makes it more convenient for preprotein transfer from Tim50 to Tim23.

The use of crystal packing as a tool to correctly predict protein–peptide interaction sites has previously been reported (Sha *et al.*, 2000 ▸). In this study, the putative presequence-binding grooves of Tim50 are occupied by helix A1 and the N-terminal proline-rich loop from neighboring monomers owing to crystal packing. The binding is primarily mediated through hydrophobic interactions and possibly generates the conformational changes within the protruding β-hairpin and the nearby helix A2. We propose that helix A1 from neighboring monomers may mimic the presequence helix to dock into the putative presequence-binding groove of Tim50 *via* hydrophobic interactions. The data suggest that the preprotein receptor Tim50 contains significant structural plasticity within the putative presequence-binding groove, particularly within the protruding β-hairpin and the nearby helix A2, to receive the presequences. As a comparison, structural plasticity has also been observed in the preprotein receptor Tom70 from the TOM complex (Li *et al.*, 2009 ▸, 2010 ▸).

## Supplementary Material

PDB reference: Tim50(164–361), 4qqf

## Figures and Tables

**Table 1:** Crystallization conditions for Tim50(164361)

Method	Hanging-drop vapor diffusion
Plate type	Hampton Research VDX 24-well plate
Temperature (K)	293
Protein concentration (mgml^1^)	15.0
Buffer composition of protein solution	10m*M* Tris pH 7.5, 150m*M* NaCl
Composition of reservoir solution	1.0*M* magnesium sulfate, 100m*M* Tris pH 7.5, 5% glycerol
Volume and ratio of drop	2 + 2l
Volume of reservoir (ml)	1

**Table 2:** Data collection and structure determination of Tim50(164361) Values in parentheses are for the highest resolution shell.

Data collection	
Diffraction source	APS beamline 22-ID
Temperature (K)	100
Detector	MAR CCD, 300mm
Crystal-to-detector distance (mm)	220
Rotation range per image ()	1
Total rotation range ()	100
Exposure time per image (s)	2
Space group	*P*4_1_2_1_2
Unit-cell parameters (, )	*a* = *b* = 164.29, *c* = 150.53, = = = 90.00
Wavelength ()	0.9500
Resolution ()	38.522.67 (2.772.672)
*R*_sym_ or *R*_merge_	0.088 (0.36)
*I*/(*I*)	28.48 (2.66)
Completeness (%)	99.79 (99.00)
Multiplicity	7.7 (3.2)
No. of collected reflections	58660 (5739)
No. of possible unique reflections	58798
Refinement	
Resolution ()	2.67
*R*_work_/*R*_free_	0.1866 (0.2704)/0.2394 (0.3274)
No. of atoms	
Protein	9305
Water	109
*B* factors (^2^)	
Protein	66.10
Water	74.5
R.m.s. deviations	
Bond lengths ()	0.009
Bond angles ()	1.38
